# Sequestered Bowel In A Atresia

**Published:** 2012-01-01

**Authors:** Lubna Ijaz

**Affiliations:** Department of Pediatric surgery, The Children’s Hospital and the Institute of Child Health Lahore, Pakistan

**Figure:**
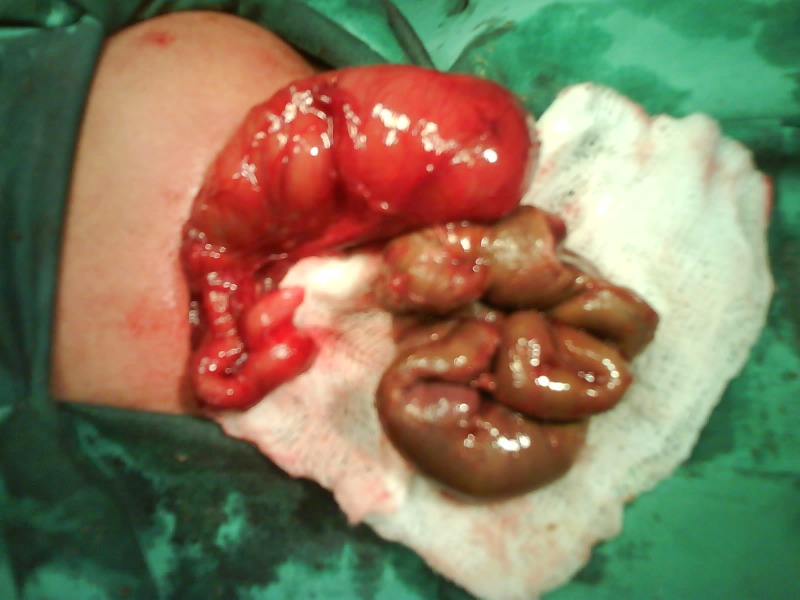
Figure 1

A 3-day-old male baby was diagnosed to have proximal small bowel atresia. At operation type-IIIa jejuno-ileal atresia (JIA) was encountered with a jumbled up sequestered bowel-loop-mass. The jejunum was 10 cm and ileum merely 1 cm. The bowel was sequestered on account of volvulus in-utero.

Atresia is derived from Greek words “a” (no) and “tresis” (orifice); and refers to congenital obstruction of intestine due to complete occlusion of the bowel lumen. JIA is most common variety of intestinal atresias followed by duodenal atresia [1,2]. The type-IIIa atresia typically develops due to in-utero volvulus of small bowel, there¬fore, usually associated with short bowel [1]. Intraute¬rine volvulus is the etiology in 25% cases of JIA. As all the cases of JIA, resulted from in-utero volvulus of small intestine, are not associated with malrotation, therefore, in-utero volvulus may develop secondary to the atresia itself where the dilated bowel can twist around its me-sentery. However, atresia can also develop secondary to volvulus even in absence of malrotation. This is especially seen in cases of meconium ileus wherein the meconium laden loop twists around its mesentery resulting in atresia, peritonitis and/or meconium cyst [3-5]. The sequestered bowel may resorpt partially or completely. Indeed complete resorption occurs in majority, however, when the event of volvulus and atresia occurs late in gestation the necrotic bowel may be present at birth.

## Footnotes

**Source of Support:** Nil

**Conflict of Interest:** None declared

